# Historical DNA from a rediscovered nineteenth-century paratype reveals genetic continuity of a Bahamian hutia (*Geocapromys ingrahami*) population

**DOI:** 10.1098/rsbl.2022.0566

**Published:** 2023-04-26

**Authors:** Michelle J. LeFebvre, Alexis M. Mychajliw, George B. Harris, Jessica A. Oswald

**Affiliations:** ^1^ Florida Museum of Natural History, University of Florida, Gainesville, FL 32611, USA; ^2^ Department of Biology, Middlebury College, Middlebury, VT 05753, USA; ^3^ Environmental Studies Program, Middlebury College, Middlebury, VT 05753, USA; ^4^ Natural History Collections, Fairbanks Museum and Planetarium, St Johnsbury, VT 05819, USA; ^5^ Department of Biology, University of Nevada, Reno, Reno, NV 89557, USA

**Keywords:** Anthropocene, Capromyinae, The Bahamas, extinction, extirpation, museum genomics

## Abstract

Past and ongoing human activities have shaped the geographical ranges and diversity of species. New genomic techniques applied to degraded samples, such as those from natural history collections, can uncover the complex evolutionary consequences of human pressures and generate baselines for interpreting magnitudes of species loss or persistence relevant to conservation. Here we integrate mitogenomic data with historical records from a recently rediscovered Bahamian hutia (*Geocapromys ingrahami*; (FMP Z02816)) specimen at the Fairbanks Museum & Planetarium (Vermont, USA) to determine when and where the specimen was collected and to place it in a phylogenetic context with specimens that both predate (palaeontological) and postdate (archaeological) human arrival in The Bahamas. We determined that this specimen was part of the same population as the named holotype specimen in 1891 on East Plana Cay (EPC). Bahamian hutia populations were widely extirpated following European colonization. Today, EPC hosts the last remaining natural Bahamian hutia population. Mitogenomic data places the focal specimen within the southern Bahamian hutia population, which is now largely restricted to EPC. The results reveal previously undocumented genetic continuity among the EPC population for at least the past 500 years, highlighting how ‘dark’ museum specimens inform new conservation-relevant understandings of diversity.

## 1. Introduction

**Figure 1.**
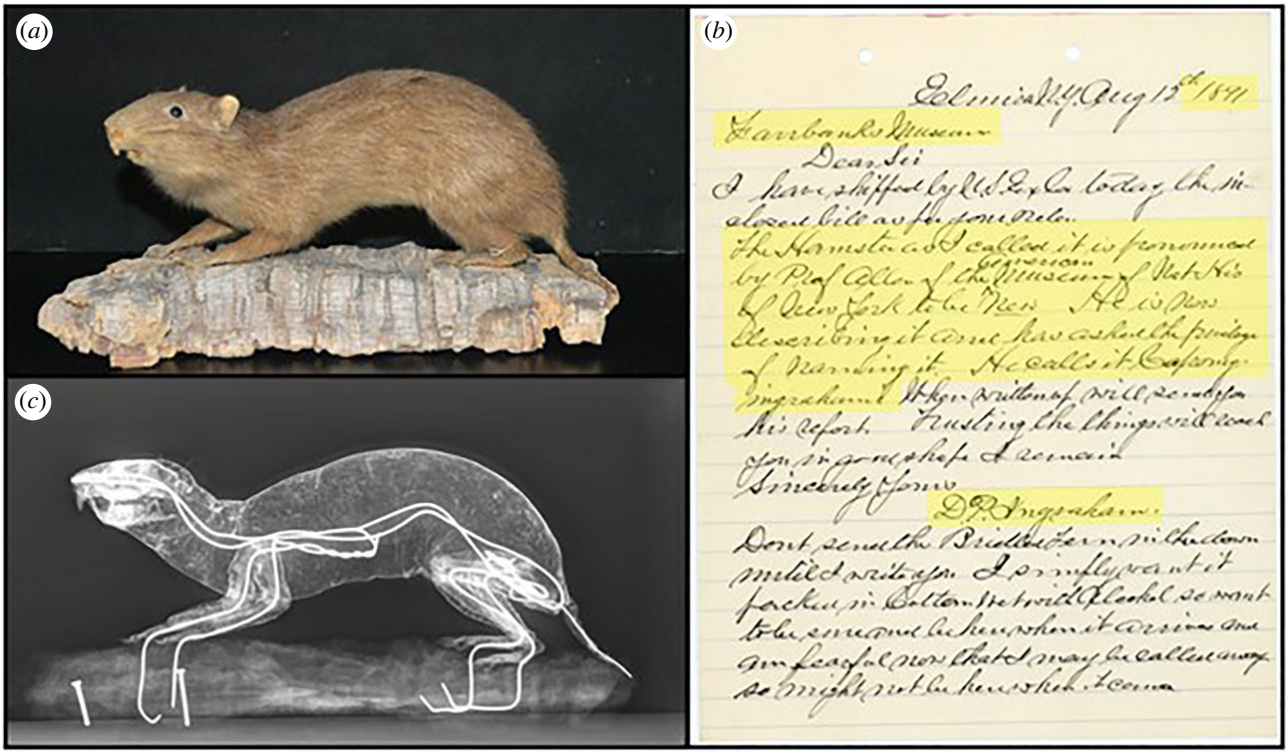
(*a*) Photograph of the individual (Z02816) Bahamian hutia (*Geocapromys ingrahami*) sampled at the FMP. (*b*) X-ray revealing the internal structure of Z02816 including the presence of some original skeletal material (skull, front and hind limbs, pelvis) and wiring used to support the taxidermy mount. (*c*) Archived correspondence of D. P. Ingraham with the Fairbanks Museum in 1891 documenting the sale of the specimen; highlighted text reads ‘*The Hamster as I called it is pronounced by Prof. Allen of the American Museum of Nat His of New York to be New. He is now describing it and has asked the privilege of naming it. He calls it Capromys ingrahami.*’. All images presented with permission of the FMP.

The application of genomic techniques to natural history collections (e.g. museum genomics [1]) is uncovering complex evolutionary and ecological histories of species [2,3], including those shaped by previously unrecognized human activities (e.g. [4,5]). As a result, millennial-scale palaeontological and archaeological collections are now being more routinely consulted as part of conservation decision-making (e.g. [6]), but there has been arguably less progress in the use of historical, centennial-scale collections [7–9]. We conducted a museum genomics study using a historical 1891 CE paratype (figure 1), allowing us to bridge the palaeontological, archaeological and neontological records of Bahamian hutia (*Geocapromys ingrahami* (J.A. Allen, 1891)) [10] diversity through time. This specimen, curated at the Fairbanks Museum & Planetarium (FMP; Vermont, USA) and collected from East Plana Cay (EPC), represents the first scientifically described population of Bahamian hutias in The Bahamas [10]. Building on existing Bahamian hutia genetic data (e.g. [11], electronic supplementary material, table S1), we present a temporally holistic phylogeny of Holocene Bahamian hutia populations, providing a series of genetic baselines that include a period of widespread extirpation between the late fifteenth and nineteenth centuries. We showcase the relevance of small-museum collections, unidentified paratypes and historical-period specimens to pressing Anthropocene conservation applications (e.g. [12]).

### (a) Bahamian hutia: a legacy of anthropogenic mass extirpation and genetic diversity loss

**Figure 2.**
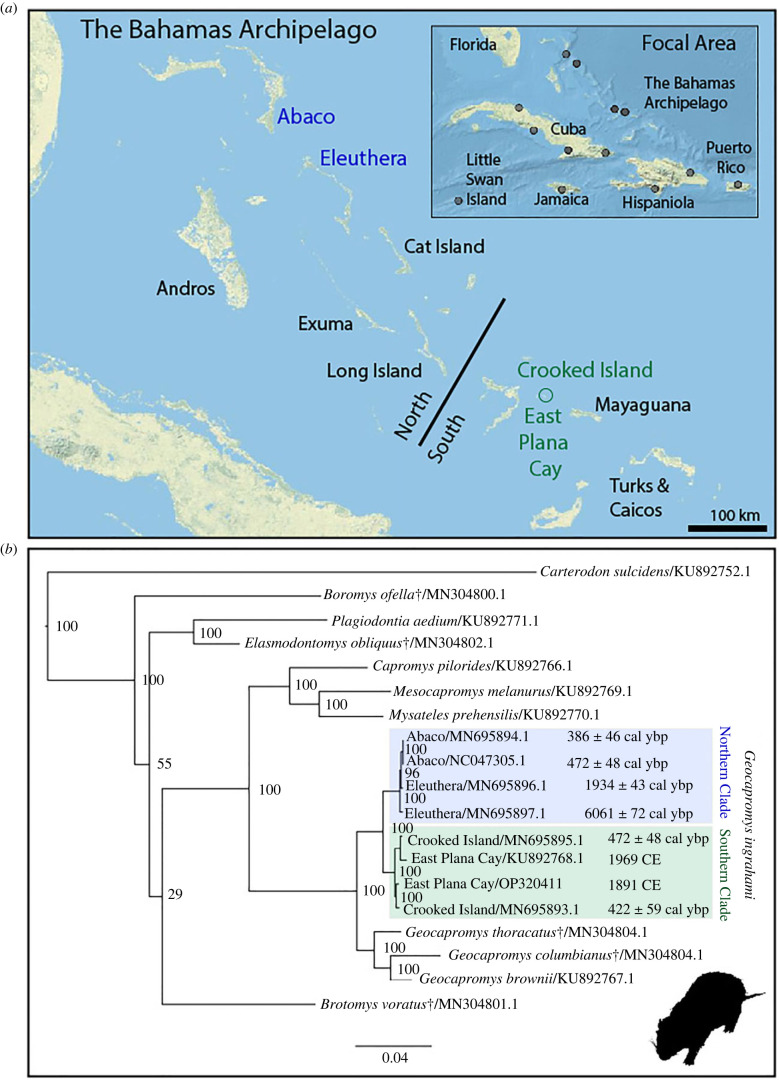
(*a*) Map of The Bahamas Archipelago featuring islands sampled and the proposed break between the Northern and Southern *Geocapromys ingrahami* clades. Inset map depicts the larger Caribbean focal area of research including The Bahamas and the Greater Antilles. Points represent locations of Caribbean rodents used in the phylogenetic analysis. Map created in ArcGIS using USA Topo Map basemap © 2013 National Geographic Society, i-cubed. (*b*) RAxML phylogeny of Capromyinae and closely related taxa based on mitochondrial genome data. Bootstrap support is indicated on the nodes. GenBank accession numbers are provided next to the taxon name. Sample age is to the right of the label and notes the two different time units. The total list of taxa and outgroup (*Trinomys* spp.) taxa can be found in the electronic supplementary material, table S1. *Geocapromys* silhouette courtesy of Phylopic.

The insular Caribbean is a biodiversity hotspot renowned for its adaptive radiations and extinctions rooted in millennial-scale human impacts following the arrival of Indigenous humans to the archipelago *ca* 6000 years ago and Europeans at the end of the fifteenth century [13,14]. Nearly half of all known hutia (endemic subfamily Capromyinae) species went extinct following human arrival, and the Bahamian hutia is now one of 11 extant hutia species [15–19]. The Bahamian hutia is the only non-volant mammal endemic to The Bahamas and is currently categorized as an IUCN vulnerable species under D2 criterion [20] (figure 2). Palaeontological and archaeological evidence shows the rodent species was once widespread across The Bahamas. Archaeological evidence further indicates the species was harvested for Indigenous subsistence and translocated across islands [21,22] without discernible widespread population declines (e.g. [11]). The arrival of Europeans resulted in the loss of a genetically distinct northern clade of Bahamian hutia, ultimately leaving only one remaining population on EPC that today is representative of a once larger southern clade (figure 2) [11].

There is sparse documentation of the historical distribution of Bahamian hutia (e.g. [23]; see also [24]) prior to first scientific collection of the species in 1891 by David P. Ingraham, a naturalist from the United States [10] (electronic supplementary material (a)). Per Ingraham's expedition notes, he only encountered hutias on EPC ([10, p.331]). While anecdotes suggest a possible population on the Samana Cays during the late 1920–1930s, there is no verifiable evidence of an early twentieth-century population outside of EPC (see [23,25]). Indeed, there are only three extant hutia populations in The Bahamas today: one native population on EPC [26,27] and one population on Warderick Wells and Little Wax Cay (Exuma Cays Land and Sea Park), respectively—each the result of conservation introductions from EPC during the 1970s and 1980s [28,29]. Conservation management of the Exumas populations is currently predicated upon limited exposure to humans, and while the EPC population is not under conservation protection, the island is documented as uninhabited by humans since at least the late nineteenth century. Using historical DNA, we targeted this period specifically with the goal of assessing the legacy effects of the species' near extinction on contemporary genetic diversity.

## 2. Materials and methods

### (a) Focal specimen

We collected dry skin fragments from the base of the tail of FMP Z02816 (figure 1) (see: permit to study specimen, electronic supplementary material (b)). Archived correspondences indicate that this specimen (accession 14285, original catalogue M21) was purchased from D. P. Ingraham by Franklin Fairbanks for $1.00 in 1891 (electronic supplementary material (a)) and listed in the FMP ledger by 1892. Mr Fairbanks then paid $2.00 for a local taxidermist, William Balch, to mount the skin (see: ‘Mr Balch's Recipe for Poison’, electronic supplementary material, figure S1).

### (b) DNA extraction and sequencing

DNA was extracted using a modified Soares *et al*. [30] protocol in a dedicated historical DNA laboratory at the Florida Museum of Natural History. Library preparation was performed with a Swift Biosciences Accel-NGS Methyl-Seq DNA kit, excluding the bisulfite conversion step (electronic supplementary material (c)). Genomic libraries were double enriched following the myBaits high sensitivity protocol (version 5) with mitochondrial baits (Arbor 303008.v5) designed for *Geocapromys*. Two 50 µl PCR reactions were prepared for the entire resuspended DNA library (30 µl total volume). The PCR product was concentrated with a vacuum centrifuge and the resultant product was used for a second round of enrichment following the same protocol as for the first round except for the PCR step. In this step, to further inhibit PCR duplicates, the two 50 µl PCR reactions for this sample were split into four 25 µl reactions. The products were combined and quantified with a Qubit^®^ 2.0 Fluorometer. This sample was quantified, cleaned and pooled at the University of Florida Interdisciplinary Center for Biotechnology Research and sequenced on an Illumina NovaSeq, S4, (¼ lane) using a 2 × 150 platform.

### (c) Data processing

Fastp 0.23.1 [31] was used to filter raw reads, remove adaptors, deduplicate and pair reads. The cleaned reads were imported into Geneious Prime (https://www.geneious.com/prime/), and the 5′ and 3′ ends were trimmed at least 8 bp to remove the adaptor sequences added in the library preparation step (swiftbiosci.com). Reads were then mapped to *Geocapromys ingrahami* (UF322691; NC047305.1) using the Geneious mapping algorithm with custom settings, 2% allowed mismatch of reads, with fine tuning set to iterate up to five times. SNP differences were evaluated by eye. A consensus sequence was produced from the read pileups based on a 75% threshold value.

### (d) Tree building

We aligned the FMP Z02816 mitochondrial genome with all recently published ancient and modern samples of the Capromyinae and *Carterodon*, with *Trinomys* serving as an outgroup (electronic supplementary material, table S1). The poorly aligning D loop (control region) was removed, and Gblocks 0.91 (Castresana 2000) was used to further remove poorly aligned regions. RAxML v. 8.2.10 (Stamatakis 2014) was used for tree building with the data partitioned by coding, non-coding and RNA. Raw pairwise distance values of the dataset were calculated in R [32] (R Core Team 2022) using the packages Pegas [33] and Ape [34].

## 3. Results and discussion

### (a) Establishing paratype status

We recovered 18 letters of correspondence from Ingraham that support the paratype status of FMP Z02816: six from FMP and 12 from the American Museum of Natural History (AMNH), with an additional two invoices from Ingraham for specimens sold to FMP (electronic supplementary material (a)). AMNH's paper catalogue agrees with Ingraham's letters describing the initial population set collected from EPC in 1891. AMNH correspondences have no mention of the FMP specimen and therefore the FMP specimen was sold separately outside of this population set; AMNH received 14 specimens and FMP received one, essentially rendering the FMP specimen ‘lost’ or unidentified. This is further corroborated by correspondence between Ingraham and FMP, which indicates he sent most of his specimens from his February 1891 collection to AMNH but held one of them out and sold it to the Fairbanks Museum as per the 5 August 1891 letter: ‘He [Allen] has asked for the entire series that I have but I will hold one for you’ (electronic supplementary material (a), Letter 4).

Within the AMNH collection, AMNH 3968 is the holotype as it is the first listed specimen in the catalogue. The AMNH museum catalogue contains Bahamian hutias across catalogue numbers 3968–3981 (original numbers 3032–3045). Four lines indicate that specimens were exchanged with three other institutions (electronic supplementary material (d)), but with little to no archival cohesion.

The ‘rediscovery’ of paratype status for this specimen illustrates the importance of preserving both specimens themselves and their related archival material as part of the scientific record, especially for smaller and non-traditional natural history and science museums. Staff at these institutions, especially in the later nineteenth and early twentieth centuries, were not necessarily trained scientists and relied on correspondence with others for identification. At institutions where collections grew out of the interests of founders in display purposes, there can be a disconnect between collection data and specimens resulting in an underutilization of the specimens' full potential.

### (b) Digitized specimen records from the global biodiversity information facility

The majority of digitized *Geocapromys ingrahami* specimen records are fossils (approx. 87%), with most held by the Harvard Museum of Comparative Zoology (MCZ) and the Florida Museum of Natural History (UF). These specimens are spread across the distributions of both northern and southern clades. The Ingraham specimens are thus the only collection from the nineteenth century. The next temporal bins represented by hutia specimens are 1932–1936, 1950–1990 and 2012–2015.

### (c) Genetic diversity and phylogeny

Sequencing generated 139 891 510 reads for FMP Z02816; filtering with fastp resulted in 67 005 358 paired reads and 50 230 733 mapped to the reference *Geocapromys ingrahami* mitochondrial genome (average read depth 134 815.4, s.d. 200 373.2; average read length 44 bp). In a phylogenetic context, the Capromyinae form a well-supported (100) clade (figure 2*b*). The clade composed of *Capromys*, *Mesocapromys*, *Mysateles* and *Geocapromys* also has maximum bootstrap support (figure 2*b*). We confirmed the previous assertion of two genetically distinct lineages of *Geocapromys ingrahami* within The Bahamas prior to and during Indigenous habitation of the region: the extinct northern lineage and the extant southern lineage, which is represented today by the population on EPC [11]. The FMP paratype specimen falls within this southern clade (figure 2). Species-level raw pairwise distance values within the genus *Geocapromys* are, on average, 4.2–6.2%, and the north and south clades within *Geocapromys ingrahami* are 1.8% divergent from each other (table 1). Within the southern clade, consisting of recent EPC and approximately 500-year-old Crooked Island samples, the FMP paratype is 0.8% divergent from the living population found on EPC and 0.3% and 0.6% divergent from the extirpated populations on Crooked Island (table 1).

**Table 1.** Raw pairwise distance values of *Geocapromys* taxa compared to the Fairbanks specimen (Z02816).

species	catalogue number	GenBank accession number	locality	age	sample type	pairwise distance
*Geocapromys ingrahami*	Fairbanks Z02816	OP320411	Southern Bahamas: East Plana Cay	1891 CE	study skin	0.000
*Geocapromys ingrahami*	USNM 395696	KU892768.1	Southern Bahamas: East Plana Cay	1969 CE	study skin	0.008
*Geocapromys ingrahami*^b^	UF 322960	MN695893.1	Southern Bahamas: Crooked Island: McKay's Bluff Cave	422 ± 59 cal ybp (Beta-502524)^c^	fossil	0.003
*Geocapromys ingrahami*^b^	UF 322949	MN695895.1	Southern Bahamas: Crooked Island: McKay's Bluff Cave	472 ± 48 cal ybp (Beta-520444)^c^	fossil	0.006
*Geocapromys ingrahami*^b^	UF 322961	NC 047305.1	Northern Bahamas: Abaco: Ralph's Cave	472 ± 48 cal ybp (Beta-502523)^c^	fossil	0.018
*Geocapromys ingrahami*^b^	UF 322959	MN695894.1	Northern Bahamas: Abaco: Hole-in-the-Wall Cave	386 ± 46 cal ybp (Beta-520445)^c^	fossil	0.018
*Geocapromys ingrahami*^b^	UF 322947	MN695897.1	Northern Bahamas: Eleuthera: Garden Cave	6061 ± 72 cal ybp (Beta-520442)^c^	fossil	0.018
*Geocapromys ingrahami*^b^	UF 322948	MN695896.1	Northern Bahamas: Eleuthera: Garden Cave,	1934 ± 43 cal ybp (Beta-520443)^c^	fossil	0.018
*Geocapromys brownii*	ZMA MAM.2388	KU892767.1	Jamaica: unknown	unknown CE	study skin	0.045
*Geocapromys thoracatus*^a^	EXEMS (28/1939/4, 1/1940/1)	MN304804.1	Little Swan Island	1939–40 CE	study skin	0.042
*Geocapromys columbianus*^a^	PMYU (210020, 210033, 210203)	MN304803.1	Cuba: Las Obas	1420 ± 79 cal ybp (Beta-214957)^	fossil	0.062

^a^ = extinct species.

^b^ = extirpated population. Radiocarbon date from.

^c^Oswald *et al.* [11] on hutia bone.

^d^Colten *et al*. [35] (2009) on associated shell calibrated using OxCal v.4.4 (Bronk Ramsey [36] 2009) and IntCal20 (Reimer [37] 2020) or MarineCal20 (Heaton *et al*. [38]), mean ± *σ* cal ybp = calibrated years before present.

Pairwise genetic distances suggest that the 131-year-old paratype FMP specimen is more similar to 500-year-old extirpated Crooked Island populations than to an EPC specimen collected in 1969 (table 1; electronic supplementary material, file S1). However, based on this one measure of diversity, it appears that now extirpated individuals from Crooked Island were 0.6% genetically divergent from each other prior to their extirpation 500 years ago (electronic supplementary material, table S1). The divergence across islands and within the southern population could have been driven by population bottlenecks or localized selection pressures linked to differential island resource availability among naturally dispersed populations (see [39])—especially considering the long isolation history of many southern Bahamian islands even during lower sea levels associated with the last glacial maximum ([40], figure 1). These same pressures could also have occurred among populations translocated by Indigenous peoples in the more recent past. Ultimately, more data are required to understand historical gene flow and bottlenecks of this species.

### (d) A small-museum specimen and museum genomics yield an initial historic conservation baseline

Our analysis of an 1891 paratype specimen begins to fill a critical temporal gap in our understanding of Bahamian hutia diversity and extirpation history. This specimen provides the first direct geographical relationship to the last remaining natural population of Bahamian hutia, linking pre-AD 1500 palaeontological and archaeological baselines with contemporary late twentieth-century neontological baselines (e.g. [21]). There are no known palaeontological or archaeological collections from the EPC population. The FMP paratype specimen provides an opportunity to assess the historical genetic diversity of the EPC population beyond the available sequenced specimen collected in 1969 (table 1).

The results indicate genetic continuity of the EPC population prior to and since Ingraham's collecting expedition, suggesting that in the absence of human pressures, introduction of non-native species, or annihilation from catastrophic storm events, an isolated population could maintain steady diversity for at least 500 years. However, it is currently unknown what the effective population size has been through time on EPC and will require sequencing of more individuals from extant and extirpated populations for diachronic comparisons with pre- and post-human contexts. We also now have a historical foundation from which to begin to generate predictive models of divergence within the EPC population given certain conservation actions. This effort will require new sampling from EPC and first-time sampling from the Exumas populations.

## Data Availability

The FMP Z02816 *Geocapromys ingrahami* mitochondrial genome is available on GenBank, accession number: OP320411. Raw reads are available on NCBI SRA: BioProject: PRJNA874730, accession: SAMN30598325. The following data and information are available as electronic supplementary material: electronic supplementary material, file S1 is the CSV of raw pairwise distance values of all Capromyinae and related taxa included in our phylogenetic dataset. Electronic supplemental material includes: (a) transcribed letters between Ingraham and the Fairbanks Museum; (b) Permit to study specimen FMP Z02816 and associated archival material; (c) DNA extraction and sequencing protocols; (d) distribution and associated catalogue numbers of the historic Ingraham-collected population. Electronic supplementary material, table S1 lists the taxa included in the phylogenetic analysis. Electronic supplementary material, figure S1 shows Mr Balch's Recipe for Poison that would likely have been applied to the study specimen. Document held by the FMP archives. The data are provided in the electronic supplementary material [41].
